# Factors associated with non-attendance at exercise-based cardiac rehabilitation

**DOI:** 10.1186/s13102-019-0125-9

**Published:** 2019-07-26

**Authors:** Sabina Borg, Birgitta Öberg, Margret Leosdottir, Daniel Lindolm, Lennart Nilsson, Maria Bäck

**Affiliations:** 1 Department of Medical and Health Sciences, Division of Physiotherapy, Linköping University, SE-581 83 Linköping, Sweden; 2 Department of Cardiology and Department of Medical and Health Sciences, Linköping University, Linköping, Sweden; 3 Department of Clinical Sciences Malmö, Faculty of Medicine, Lund University, Malmö, Sweden; 4 Department of Cardiology, Skåne University Hospital, Malmö, Sweden; 5 Department of Medical Sciences, Cardiology, Uppsala University; and Uppsala Clinical Research Center, Uppsala, Sweden; 6 Department of Medical and Health Sciences, Division of Cardiovascular Medicine, Linköping University, Linköping, Sweden; 7 Department of Occupational Therapy and Physiotherapy, Sahlgrenska University Hospital, Gothenburg, Sweden

**Keywords:** Acute myocardial infarction, Coronary artery disease, Exercise-based cardiac rehabilitation, Non-attendance, Physiotherapy, Secondary prevention

## Abstract

**Background:**

Despite its well-established positive effects, exercise-based cardiac rehabilitation (exCR) is underused in patients following an acute myocardial infarction (AMI). The aim of the study was to identify factors associated with non-attendance at exCR in patients post-AMI in a large Swedish cohort.

**Methods:**

A total of 31,297 patients who have suffered an AMI, mean age 62.4 ± 4 years, were included from the SWEDEHEART registry during the years 2010–2016. Comparisons between attenders and non-attenders at exCR were done at baseline for the following variables: age, sex, body mass index, occupational status, smoking, previous diseases, type of index cardiac event and intervention, and left ventricular function. Distance of residence from the hospital and type of hospital were added as structural variables in logistic regression analyses, with non-attendance at exCR at one-year follow-up as dependent, and with individual and structural variables as independent variables.

**Results:**

In total, 16,214 (52%) of the patients did not attend exCR. The strongest predictor for non-attendance was distance to the exCR centre (OR 1.75 [95% CI: 1.64–1.86]). Other predictors for non-attendance included smoking, history of stroke, percutaneous coronary intervention (PCI), coronary artery bypass grafting (CABG), AMI or diabetes, male sex, being retired vs. being employed, and being followed-up at a county hospital. Patients with ST-elevation myocardial infarction (STEMI) and those intervened with PCI or CABG were more likely to attend exCR.

**Conclusions:**

A distance greater than 16 km was associated with increased probability of non-attendance at exCR, as were smoking, a higher burden of comorbidities, and male sex. A better understanding of individual and structural factors can support the development of future rehabilitation services.

## Background

Coronary artery disease (CAD), including acute myocardial infarction (AMI), is the main cause of disability and death in developed countries placing a major burden on the healthcare systems and the economy [1]. In recent decades, mortality rates from CAD have fallen, mainly due to improved medical treatment and better control of cardiovascular risk factors, and this has led to a larger number of patients in need of secondary prevention [1]. Meta-analyses have confirmed the positive effects of exercise-based cardiac rehabilitation (exCR), in terms of a marked reduction in cardiovascular mortality, a reduced risk of hospital readmission [2, 3], and favourable effects on cardiovascular risk factors, aerobic capacity, anxiety and depression [2–4]. Therefore, exCR is often identified as the cornerstone of CR [5, 6] and has been given a class I recommendation in guidelines published by the European Society of Cardiology [6] and the American Heart Association [5]. In order to achieve the well-established positive health benefits of exCR, uptake and adherence to exCR programmes are important [6].

Despite beneficial effects and international recommendations, exCR continues to be widely underused with overall participation rates in recent decades of about 40% [7]. Suboptimal participation rates at exCR are also a concern in Sweden. According to the latest report from the national quality registry Secondary Prevention After Intensive Heart Care Unit (SEPHIA) [8], which is part of the SWEDEHEART registry, only 19% of patients had participated in an exCR programme during the first year after an AMI in, with a large inter-hospital variation, indicating a great potential for improvement.

A Cochrane review has concluded that there is weak evidence of the effect of current interventions that aim to increase uptake and adherence to exCR programmes [9]. Factors related to non-attendance at centre-based exCR are usually categorized into sociodemographic, medical, personal and healthcare-related factors [10–14]. However, contextual aspects that are important for attending exCR, such as accessibility issues, comorbidities distribution and patient referral routines, may differ between countries, which highlights the need to study national perspectives. The aim of the study presented here was to identify factors associated with non-attendance at exCR in patients post-AMI in a large Swedish cohort.

## Methods

### Study design

A retrospective, registry-based cohort study.

### Setting

The SWEDEHEART registry provides continuous information on patient care needs, treatments and treatment outcomes. The purpose of SWEDEHEART is to register changes in the quality and content of patient care over time, to provide decision support, and to support continuous improvement. The coverage of SWEDEHEART is high, with all Swedish hospitals reporting to the registry. The SWEDEHEART registry consists of several sections, one of which is the Register of Information and Knowledge about Swedish Heart Intensive Care Admissions (RIKS-HIA), which describes the acute care of AMI. Another section is SEPHIA, which describes performance in secondary prevention care after AMI, including participation in exCR, treatment goal fulfilment, and cardiovascular and psychological status. SEPHIA comprises post-AMI patients younger than 75 years. More than 7000 new patients are registered every year. Data registered in RIKS-HIA are collected by healthcare providers during hospitalization. SEPHIA has information from two follow-up visits during the first year after the AMI (visit 1, 6–10 weeks post-AMI, and visit 2, 12–14 months post-AMI).

### Study population

**Fig. 1:**
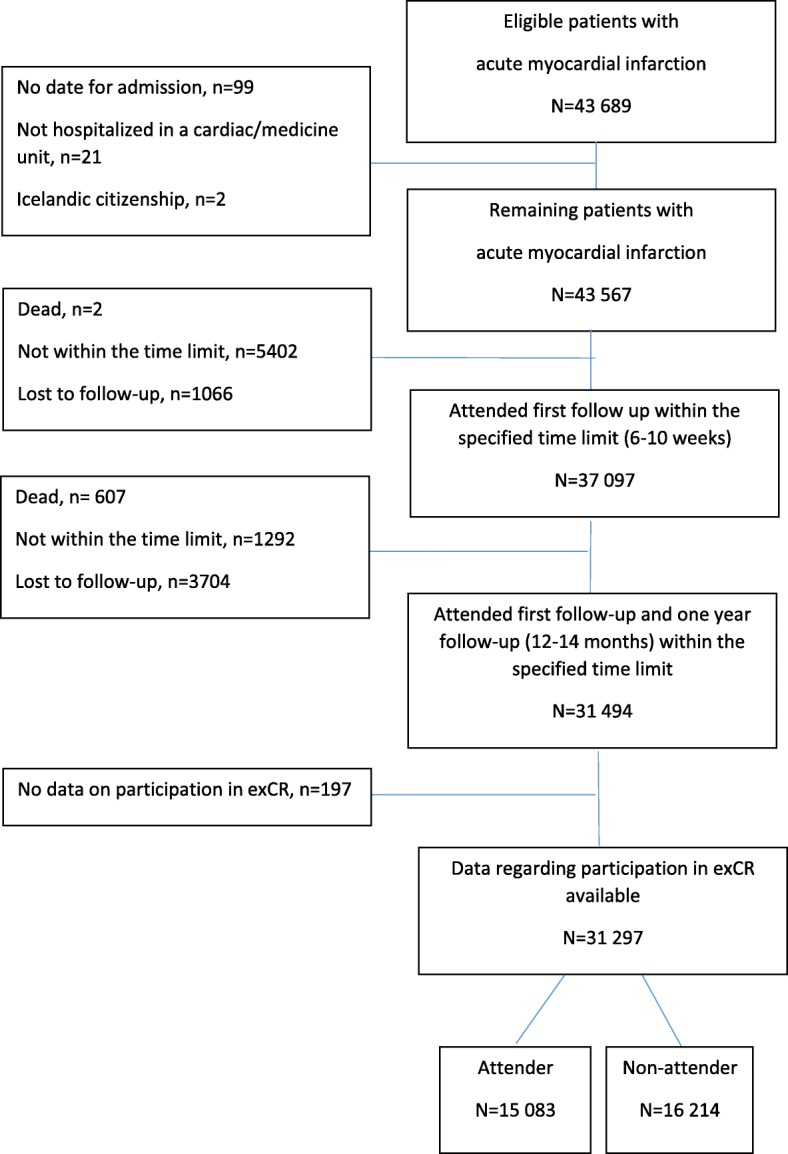
Flowchart of included patients. Flowchart of patients following an acute myocardial infarction registered in SEPHIA and RIKS-HIA between 2010 and 2016

The study population was defined as patients registered in SEPHIA (which comprises approximately 70% of all Swedish patients with AMI), younger than 75 years, included in RIKS-HIA, and alive at 2 months post-AMI. Out of 43,689 eligible patients, a total of 31,297 were included. These had been monitored in SEPHIA and RIKS-HIA for six consecutive years (2010–2016). Figure 1 shows further details. Inclusion criteria were: individuals registered in SEPHIA with an index diagnosis of AMI and for whom follow-up visit 1 and 2 had occurred during the date range, 1 January 2010 to 31 December 2016.

### Variables

Table 1 shows an overview of included variables. The following **individual variables** were obtained from the RIKS-HIA registry at hospital admission or discharge (referred to here as “baseline”). The variables were self-reported or retrieved from patient records.

**Table 1:** Included variables

Individual variables	Structural variables
Demographic variables	Type of hospital
Body mass index	Distance to hospital
Occupational status	
Smoking	
Previous diseases	
Index event and intervention	
Left ventricular function	
Medication	

#### Attendance at exCR

Attendance at exCR was defined by the self-reported variable “participated in an exercise training programme”, registered at visit 2.

#### Demographic variables

Age (years), sex (male/female).

#### Body mass index

Body mass index (BMI) was calculated as the weight in kilograms divided by the height in metres squared (kg/m^2^).

#### Occupational status

The variable included four different categories: employed, retired, on sick leave, and unemployed/student/other.

#### Smoking

The variable contained three different categories: never smoked, former smoker (duration longer than 1 month), and current smoker.

#### Previous diseases

Previous diseases were defined as a history of CAD in terms of AMI, percutaneous coronary intervention (PCI) or coronary artery bypass grafting (CABG), diabetes, hypertension, chronic heart failure, and stroke.

#### Index event and intervention

Type of myocardial infarction was defined as ST-elevation myocardial infarction (STEMI) or non-ST-elevation myocardial infarction (NSTEMI), while the type of intervention was defined as PCI or CABG.

#### Left ventricular function

Left ventricular function was defined as normal (ejection fraction (EF) > 50%), slightly reduced (EF 40–49%) or moderate/severely reduced (EF < 39%).

#### Medication

Current AMI medication included: angiotensin-converting enzyme (ACE), angiotensin receptor blocker (ARB), acetylsalicylic acid (ASA), anti-platelets and anticoagulants, beta-blockers and statins.

The following **structural variables** were included:

#### Type of hospital

Data of all registered hospitals at visit 2 were categorized into three groups: university hospital, county hospital, and district hospital. This formed the structural variable “type of hospital”.

#### Distance to hospital

The patient’s driving distance to the hospital was estimated using a custom interface to the OpenStreetMap Routing Machine. The centroid of each patient’s postal code area served as a proxy for their address, from which the route to the nearest hospital was calculated [15].

### Statistical methods

Data analyses were conducted using SPSS Version 24 for Windows (IBM Corp. Armonk, NY) and R Version 3.4.4 (R Foundation for Statistical Computing, Vienna, Austria, 2018). Continuous variables are presented as means and standard deviations (SD) for normally distributed variables, and medians and interquartile ranges for non-normally distributed variables. Categorical variables are reported by numbers and proportions (%). Comparisons between attenders and non-attenders at exCR were carried out at baseline for each individual variable using Student’s T-test or the Wilcoxon test for continuous variables, and the chi-squared test for categorical variables as appropriate.

Missing values in at least one variable was present in about 15% of observations, although infrequent when looking per variable. Therefore, imputation by chained tree-ensembles was performed (as implemented in the missRanger R package, setting the maximum number of chaining iterations to 10 (the default value)).

The distribution of driving distance was right-skewed, and it was therefore log-transformed before analysis. The unadjusted association between driving distance and non-attendance at exCR was assessed by a spline plot, entering driving distance as a restricted cubic spline in an unadjusted logistic regression model.

The first stage of the multivariable analyses was to fit a logistic regression model that included age, sex, BMI, occupational status, smoking status, previous diseases, type of index cardiac event, type of intervention, and type of hospital at follow-up visit 2 as independent variables. This fitting procedure used non-attendance at exCR at follow-up visit 2 as dependent variable. The second stage of the analysis was to fit an additional model that also included driving distance. All continuous variables were entered as restricted cubic splines to account for possible non-linear associations with outcome. The results are presented as odds ratios (OR) and 95% confidence intervals (CI). The Wald statistic (the number of degrees of freedom [df] for each variable subtracted from its chi-squared value) was used as a variable importance metric.

## Results

### Demographics

Table 2 shows baseline characteristics for the variables. In total, 16,214 (52%) of patients post-AMI did not attend exCR. Non-attenders were older, more often retired, had more previous diseases (diabetes, AMI, PCI, CABG, chronic heart failure, stroke), higher BMI, lower left ventricular function, and were more often smokers (all *p*-values < 0.009) than attenders.

**Table 2:** Baseline demographics for attenders and non-attenders at exercise-based cardiac rehabilitation, *n* = 31 297

Attending exercise-based CR	No	Yes	*p*-value	missing, %
	*n* = 16 214	*n* = 15 083		
Age (years), mean (SD)	62.9 (8.3)	62.0 (8.4)	< 0.001^a^	0.0
BMI (kg/m^2^), mean (SD)	27.8 (4.5)	27.7 (4.3)	0.009^a^	3.6
Sex, n (%)			0.058^b^	0.0
- Men	12212 (75.3)	11219 (74.4)		
- Women	4002 (24.7)	3864 (25.6)		
Occupational status, n (%)			< 0.001^b^	5.5
- Employed	6152 (40.1)	6711 (47.2)		
- Retired	8321 (54.2)	6810 (47.9)		
- Sick leave	431 (2.8)	344 (2.4)		
- Unemployed, student, other	435 (2.8)	365 (2.6)		
Smoking status, n (%)			< 0.001^b^	2.2
- Never smoked	5002 (31.5)	5563 (37.8)		
- Ex- smoker > 1 month	5615 (35.4)	5455 (37.0)		
- Smoker	5252 (33.1)	3718 (25.2)		
Previous diseases, n (%)				
- AMI	2853 (17.7)	1718 (11.4)	< 0.001^b^	0.4
- PCI	2339 (14.5)	1407 (9.4)	< 0.001^b^	0.4
- CABG	1100 (6.8)	576 (3.8)	< 0.001^b^	0.2
- Diabetes	3150 (19.5)	2326 (15.5)	< 0.001^b^	0.2
- Hypertension	7049 (43.8)	6406 (42.7)	0.057^b^	0.6
- Chronic heart failure	625 (4.0)	386 (2.6)	< 0.001^b^	3.3
- Stroke	736 (4.6)	431 (2.9)	< 0.001^b^	0.3
Type of index cardiac event, n (%)			< 0.001^b^	0.0
- STEMI	5986 (36.9)	6279 (41.6)		
- NSTEMI	10228 (63.1)	8804 (58.4)		
Type of index cardiac intervention, n (%)				
- PCI	13204 (81.4)	12588 (83.5)	< 0.001^b^	0.0
- CABG	644 (4.0)	878 (5.8)	< 0.001^b^	0.0
Left ventricular function, n (%)			< 0.001^b^	13.0
- Normal	9288 (66.5)	9091 (68.5)		
- Lightly reduced	2767 (19.8)	2648 (19.9)		
- Moderate/severely reduced	1903 (13.6)	1535 (11.6)		
Medication, n (%)				
- ACE	10979 (67.7)	10313 (68.4)	0.025^b^	0.1
- ARB	2697 (16.6)	2613 (17.3)	0.108^b^	0.1
- Anticoagulants	1014 (6.3)	805 (5.4)	0.001^b^	0.1
- Other platelet inhibitors	14951 (92.2)	13857 (91.9)	< 0.01^b^	0.1
- ASA	15623 (96.4)	14655 (97.2)	< 0.001^b^	< 0.1
- Beta-blockers	14744 (91.0)	13889 (92.1)	< 0.001^b^	< 0.1
Statins	15691 (96.8)	14809 (98.2)	< 0.001^b^	< 0.1
Distance to hospital, median (IQR)	11.3 (4.4–25.3)	18.3 (5.8–34.3)	< 0.001^c^	1.6
Type of hospital, n (%)			< 0.001^b^	0.0
- University hospital	3462 (21.4)	3982 (26.4)		
- County hospital	7079 (43.7)	5698 (37.8)		
- District hospital	5673 (35.0)	5403 (35.8)		

*CR* cardiac rehabilitation, *BMI* body mass index, *AMI* acute myocardial infarction, *PCI* percutaneous coronary intervention, *STEMI* ST-elevation myocardial infarction, *NSTEMI* non-ST-elevation myocardial infarction, *CABG* coronary artery bypass grafting, *ACE* angiotensin-converting enzyme, *ARB* angiotensin receptor blocker, *ASA* acetylsalicylic acid

^a^Student’s t-test

^b^Chi^2^- test

^c^Wilcoxon test

### Unadjusted association

**Fig. 2:**
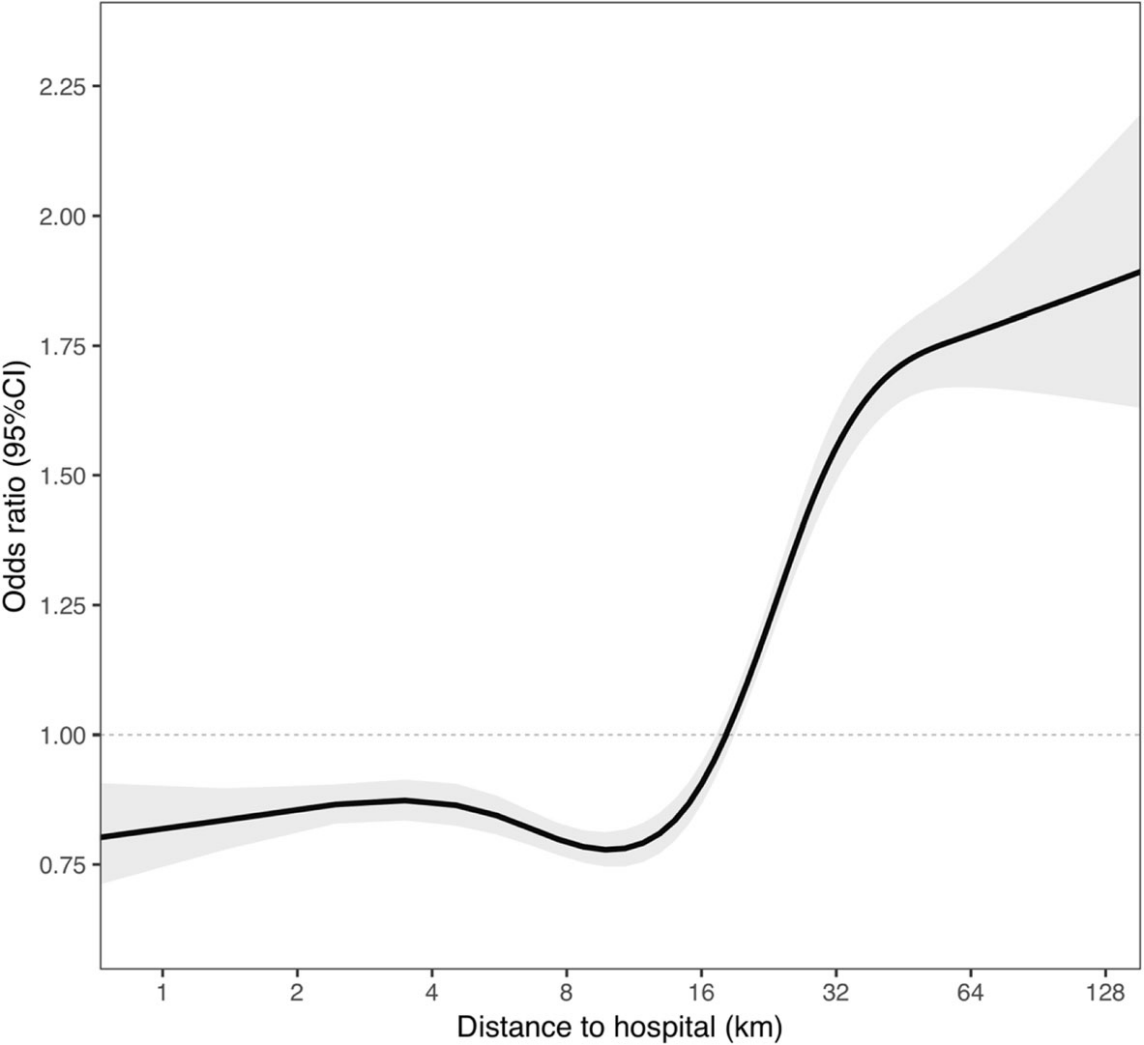
Association between distance to hospital and non-attendance at exCR. Figure 2 shows the unadjusted association between driving distance from the hospital and non-attendance at exercise-based cardiac rehabilitation

Figure 2 shows the unadjusted association between driving distance from the hospital and non-attendance at exCR, where distance has been modelled using a restricted cubic spline to account for non-linearities. Figure 2 shows a non-linear relationship, in which distance over 16 km is associated with an increased probability of non-attendance at exCR.

### Logistic regression models

**Fig. 3:**
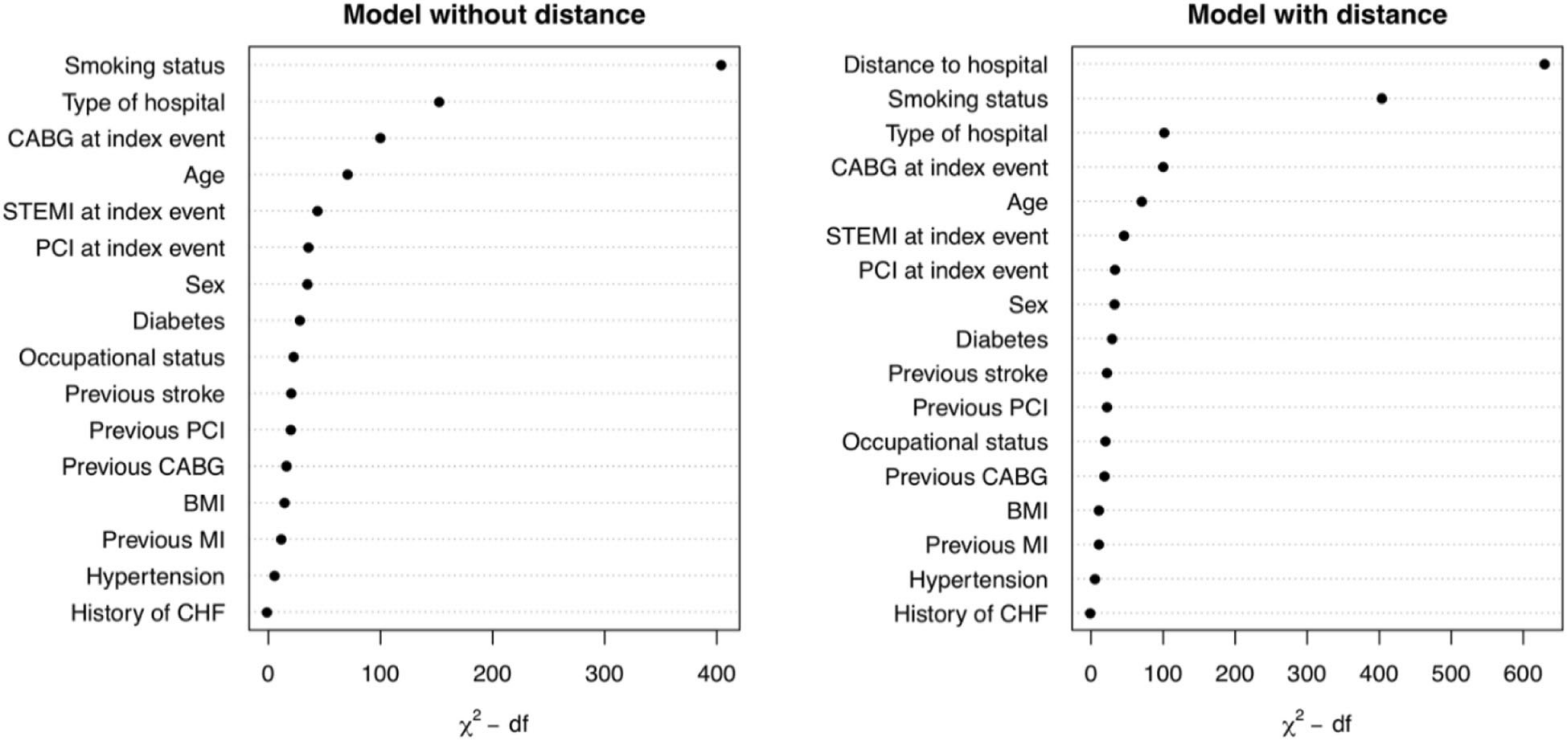
Variable importance in a model with and without distance to hospital as an independent variable. Figure 3 shows the importance of the variables in the regression model, with and without driving distance as an independent variable, for non-attendance at exercise-based cardiac rehabilitation

Figure 3 illustrates the importance of the variables in the regression model, with and without driving distance as an independent variable. The model as a whole explained 5.1% of the variance without driving distance included as an independent variable, and 7.6% of the variance with driving distance included as an independent variable. As such, the amount of variation in non-attendance at exCR explained by the model is markedly higher when distance to the hospital is included as an independent variable.

The results of the logistic regression model with driving distance included as an independent variable are presented in Table 3. The strongest independent variable that predicts non-attendance at exCR was distance to hospital (OR 1.75 [95% CI: 1.64–1.86] comparing upper and lower quartiles). Other predictors for non-attendance were current smoking, a history of AMI, PCI, CABG, diabetes or stroke, male sex, being retired vs. being employed, having NSTEMI as the index cardiac event, and not having undergone an intervention (PCI or CABG). Also, patients belonging to a county hospital were less likely to attend exCR than those belonging to a university hospital or a district hospital.

**Table 3:** Logistic regression model with distance to CR-centre included as an independent variable

Variable	OR	Lower 95%	Upper 95%
Age	1.00	0.90	1.10
BMI	1.05	0.99	1.11
Sex (female vs male)	0.85	0.80	0.90
Occupational status (employed vs retired)	0.86	0.80	0.93
Occupational status (sick leave vs retired)	1.05	0.89	1.23
Occupational status (unemployed/student/other vs retired	1.02	0.87	1.20
Smoking status (smoker vs ex-smoker)	1.63	1.54	1.74
Smoking status (never smoked vs ex-smoker)	0.91	0.86	0.96
Previous diseases (yes vs no)			
Diabetes	1.20	1.13	1.28
Hypertension	0.94	0.89	0.98
Chronic heart failure	1.01	0.88	1.16
Stroke	1.37	1.21	1.55
Acute myocardial infarction (AMI)	1.19	1.08	1.31
Percutaneous coronary intervention (PCI)	1.28	1.16	1.42
Coronary artery bypass grafting (CABG)	1.31	1.16	1.48
Type of index cardiac event and intervention			
STEMI vs NSTEMI	0.84	0.80	0.88
PCI (yes vs no)	0.81	0.76	0.87
CABG (yes vs no)	0.55	0.49	0.62
Distance to hospital (km)	1.74	1.62	1.86
Type of hospital (university hospital vs county hospital)	0.79	0.75	0.84
Type of hospital (district hospital vs county hospital)	0.78	0.74	0.82

*STEMI* ST-elevation myocardial infarction, *NSTEMI* non-ST-elevation myocardial infarction

## Discussion

This study adds to the existing literature by examining both individual and structural factors, such as type of hospital and distance to the CR centre, that influence attendance at exCR in a large national cohort of patients post-AMI covering a six-year period. The strongest predictor for non-attendance at exCR was distance to hospital, and the unadjusted association showed a non-linear relationship in which a distance greater than approximately 16 km was associated with a substantially increased risk of non-attendance at exCR.

Proximity to a CR centre has repeatedly been found to play an important role for attendance at exCR [11, 13, 14], but few studies have used objective geographic indicators to measure distance to the CR centre [16–18] as we have. Suaya et al. [19] studied a large sample size of older patients with CAD, using the patients’ postal codes to measure distance to the nearest CR centre. They showed that distance was an important predictor for multifactorial CR utilization. However, the study did not specifically investigate the effect of distance on uptake to exCR programmes [19]. The distance of 16 km that we have determined as the limit for increased probability for non-attendance at exCR can be compared with the results by Brual et al. [16], who showed that a driving time of 60 min or more to the nearest CR centre was associated with decreased CR referral and enrollment. Another study found that enrollment in CR programmes was lower for those who lived further than 50 km from the CR centre [17]. These differences may be the result of accessibility issues such as heavy traffic and availability of public transportation, or the result of organizational aspects concerning where exCR is delivered. We show here that patients who were followed by university hospitals and district hospitals had higher attendance at exCR than those who were followed by county hospitals, which suggests that there are advantages in uptake for both large, highly specialized hospitals and for the smallest hospitals.

Distance to the CR centre is a complex concept and can be measured both as distance in kilometres and in driving time. Furthermore, it may be perceived differently in urban and rural areas. Therefore, a broader understanding of this field requires more studies of objectively measured aspects of the association between distance and non-attendance at exCR in different contexts. Other aspects that require more study are how the type of hospital affects perceptions of distance, and the effects of, for example, different models of care, and the expertise of the healthcare providers.

In an attempt to overcome geographical barriers and widen access to exCR, changes to the organization of exCR are being considered. Home-based programmes and eHealth solutions are being discussed as alternative models to centre-based exCR [20–23], as is also the possibility of providing CR centres closer to patients’ homes through cooperation with smaller healthcare centres. Driving time and distance are important factors that influence patients’ decisions to choose home-based exCR over hospital-based exCR [23]. We suggest that greater tailoring of exercise programmes in liaison with the patient is needed, and that an insight into patient perspectives is important for a deeper understanding of aspects that affect attendance at exCR. Psychological support and guidance provided by the physiotherapist at the CR centre in learning the appropriate level of effort during exercise is important in reducing the fear of exercise after an AMI [24].

The individual factors related to non-attendance at centre-based exCR in a Swedish context that we have identified agree well with the results of previous studies from other countries [10–14]. Similar to other studies, we found that the risk of non-attendance at exCR was higher for individuals with a higher burden of comorbidities [11, 13, 25] and for smokers [13]. In contrast, it was lower for employed individuals [13]. Unlike most previous studies, which clearly demonstrate lower CR uptake in women [11, 13, 25], we found that female sex was associated with higher attendance at exCR. Previous literature has examined barriers for participation in CR programmes in women, including family obligations, caretaking responsibilities and multiple role conflicts [26]. The fact that Sweden is one of the world’s most gender-equal countries may play a role in explaining our findings.

### Strengths and limitations

A registry-based study mirrors the real-world population in a large sample, which increases the generalizability of the results. The large amount of data has given us the opportunity to analyze structural factors and obtain new information in this field. However, the population is not completely unselected, since only approximately 70% of patients post-AMI are registered in SEPHIA. Patients who have suffered an AMI who are not recorded in the SEPHIA registry have a higher prevalence of cardiovascular risk factors and a greater history of cardiovascular disease than the patients included in the study presented here, and it is thus possible that we have investigated a selection of patients more prone to secondary prevention.

The SWEDEHEART registry has high coverage, and the data are entered in the registry with high accuracy due to the use of highly standardized data-collection procedures. This ensures a high validity of the data. However, participation in exCR is based on patients’ self-reported information, not on an objective measure, which is a limitation in the study. Another limitation is that we have studied only patients younger than 75 years, which was the limit of information in the SEPHIA registry at the time. High age is a barrier for uptake in exCR [10, 13, 25], which declines significantly after age 70 years [27].

In the present study, the regression model explained 7.6% of the variance when distance to the CR-centre was included as an independent variable. It is possible that the amount of variance in non-attendance at exCR that the model explains is larger when more variables are included, such as culture/ethnical aspects, personal factors (such as lack of disease awareness and low self-efficacy [11, 14, 25]), socioeconomic status [11, 13, 25], and civil status [13].

The SEPHIA registry does not provide information about physical activity level and physical fitness at baseline, and it is thus not possible to control for former exercise habits.

## Conclusions

This study contributes important new knowledge and shows that distance to the CR centre (measured using patients’ postal codes) is a significant predictor for non-attendance at exCR in a large national cohort. The individual factors associated with non-attendance at exCR that we have identified agree with those identified in previous studies, with the exception that female sex was associated with higher attendance at exCR. Improving access to exCR is important and should be given a high priority in order to secure equal healthcare for all patients post-AMI. Understanding and awareness of the individual and structural factors that are for uptake of and adherence to exCR are necessary in order to design, adapt and individualize actions aimed to improve participation in these programmes.

## Data Availability

The data that support the findings of this study are available from the SWEDEHEART registry, but restrictions apply to the availability of these data, which were used under license for the current study, and are thus not publicly available. The data are, however, available from the authors upon reasonable request and with permission of the SWEDEHEART registry.

## Abbreviations

ACE: Angiotensin-converting enzyme
AMI: Acute myocardial infarction
ARB: Angiotensin receptor blocker
ASA: Acetylsalicylic acid
BMI: Body mass index
CABG: Coronary artery bypass grafting
CAD: Coronary artery disease
CR: Cardiac rehabilitation
EF: Ejection fraction
exCR: exercise-based cardiac rehabilitation
NSTEMI: Non-ST-elevation myocardial infarction
PCI: Percutaneous coronary intervention
STEMI: ST-elevation myocardial infarction

## References

[1] Mozaffarian D, Benjamin EJ, Go AS, Arnett DK, Blaha MJ, Cushman M, Das SR, de Ferranti S, Despres JP, Fullerton HJ (2016). Executive summary: heart disease and stroke statistics--2016 update: a report from the American Heart Association. Circulation.

[2] Anderson L, Thompson DR, Oldridge N, Zwisler AD, Rees K, Martin N, Taylor RS. Exercise-based cardiac rehabilitation for coronary heart disease. Cochrane Database Syst Rev. 2016;(1):CD001800. doi: 10.1002/14651858. 10.1002/14651858.CD001800.pub3 PMC6491180 26730878

[3] Lawler PR, Filion KB, Eisenberg MJ (2011). Efficacy of exercise-based cardiac rehabilitation post-myocardial infarction: a systematic review and meta-analysis of randomized controlled trials. Am Heart J.

[4] Verschueren S, Eskes AM, Maaskant JM, Roest AM, Latour CHM, Op Reimer WS (2018). The effect of exercise therapy on depressive and anxious symptoms in patients with ischemic heart disease: a systematic review. J Psychosom Res.

[5] Balady GJ, Williams MA, Ades PA, Bittner V, Comoss P, Foody JM, Franklin B, Sanderson B, Southard D, American Heart Association Exercise CR, et al. Core components of cardiac rehabilitation/secondary prevention programs: 2007 update: a scientific statement from the American Heart Association Exercise, Cardiac Rehabilitation, and Prevention Committee, the Council on Clinical Cardiology; the Councils on Cardiovascular Nursing, Epidemiology and Prevention, and Nutrition, Physical Activity, and Metabolism; and the American Association of Cardiovascular and Pulmonary Rehabilitation. Circulation, 2682. 2007;115(20):2675–82. 10.1161/CIRCULATIONAHA.106.180945 17513578

[6] Piepoli MF, Corra U, Adamopoulos S, Benzer W, Bjarnason-Wehrens B, Cupples M, Dendale P, Doherty P, Gaita D, Hofer S (2014). Secondary prevention in the clinical management of patients with cardiovascular diseases. Core components, standards and outcome measures for referral and delivery: a policy statement from the cardiac rehabilitation section of the European Association for Cardiovascular Prevention & Rehabilitation. Endorsed by the Committee for Practice Guidelines of the European Society of Cardiology. Eur J Prev Cardiol.

[7] Kotseva Kornelia, Wood David, De Bacquer Dirk (2018). Determinants of participation and risk factor control according to attendance in cardiac rehabilitation programmes in coronary patients in Europe: EUROASPIRE IV survey. European Journal of Preventive Cardiology.

[8] Jernberg T (2018). SWEDEHEART annual report 2018. Matador kommunikation AB.

[9] Karmali KN, Davies P, Taylor F, Beswick A, Martin N, Ebrahim S (2014). Promoting patient uptake and adherence in cardiac rehabilitation. Cochrane Database Syst Rev.

[10] Taylor GH, Wilson SL, Sharp J (2011). Medical, psychological, and sociodemographic factors associated with adherence to cardiac rehabilitation programs: a systematic review. J Cardiovasc Nurs.

[11] Balady GJ, Ades PA, Bittner VA, Franklin BA, Gordon NF, Thomas RJ, Tomaselli GF, Yancy CW, American Heart Association Science Advisory Coordinating C (2011). Referral, enrollment, and delivery of cardiac rehabilitation/secondary prevention programs at clinical centers and beyond: a presidential advisory from the American Heart Association. Circulation.

[12] Clark AM, King-Shier KM, Spaling MA, Duncan AS, Stone JA, Jaglal SB, Thompson DR, Angus JE (2013). Factors influencing participation in cardiac rehabilitation programmes after referral and initial attendance: qualitative systematic review and meta-synthesis. Clin Rehabil.

[13] Ruano-Ravina A, Pena-Gil C, Abu-Assi E, Raposeiras S, van’t Hof A, Meindersma E, Bossano Prescott EI, Gonzalez-Juanatey JR (2016). Participation and adherence to cardiac rehabilitation programs. a systematic review. Int J Cardiol.

[14] Campkin LM, Boyd JM, Campbell DJT (2017). Coronary artery disease patient perspectives on exercise participation. J Cardiopulm Rehabil Prev.

[15] Lindholm D, James S, Lagerqvist B, Hlatky MA, Varenhorst C. New method for assessing the effect of driving distance to hospital care: using OpenStreetMap routing in cardiovascular research. Circ Cardiovasc Qual Outcomes. 2017;10(9). doi: 10.1161/CIRCOUTCOMES.117.003850. 10.1161/CIRCOUTCOMES.117.003850 28844994

[16] Brual Janette, Gravely-Witte Shannon, Suskin Neville, Stewart Donna E, Macpherson Alison, Grace Sherry L (2010). Drive time to cardiac rehabilitation: at what point does it affect utilization?. International Journal of Health Geographics.

[17] Chamosa S, Alarcon JA, Dorronsoro M, Madruga FJ, Barrera J, Arrazola X, de la Cuesta P, Alkiza ME, Begiristain JM, Carrera I (2015). Predictors of enrollment in cardiac rehabilitation programs in Spain. J Cardiopulm Rehabil Prev.

[18] Leung YW, Brual J, Macpherson A, Grace SL (2010). Geographic issues in cardiac rehabilitation utilization: a narrative review. Health Place.

[19] Suaya JA, Shepard DS, Normand SL, Ades PA, Prottas J, Stason WB (2007). Use of cardiac rehabilitation by Medicare beneficiaries after myocardial infarction or coronary bypass surgery. Circulation.

[20] Devi R, Singh SJ, Powell J, Fulton EA, Igbinedion E, Rees K. Internet-based interventions for the secondary prevention of coronary heart disease. Cochrane Database Syst Rev. 2015;(12):CD009386. doi: 10.1002/14651858.CD009386.pub2. 10.1002/14651858.CD009386.pub2 PMC10819100 26691216

[21] Huang K, Liu W, He D, Huang B, Xiao D, Peng Y, He Y, Hu H, Chen M, Huang D (2015). Telehealth interventions versus center-based cardiac rehabilitation of coronary artery disease: a systematic review and meta-analysis. Eur J Prev Cardiol.

[22] Anderson L, Sharp GA, Norton RJ, Dalal H, Dean SG, Jolly K, Cowie A, Zawada A, Taylor RS (2017). Home-based versus Centre-based cardiac rehabilitation. Cochrane Database Syst Rev.

[23] Grace SL, McDonald J, Fishman D, Caruso V (2005). Patient preferences for home-based versus hospital-based cardiac rehabilitation. J Cardpulm Rehabil.

[24] Back M, Oberg B, Krevers B (2017). Important aspects in relation to patients’ attendance at exercise-based cardiac rehabilitation - facilitators, barriers and physiotherapist's role: a qualitative study. BMC Cardiovasc Disord.

[25] Neubeck L, Freedman SB, Clark AM, Briffa T, Bauman A, Redfern J (2012). Participating in cardiac rehabilitation: a systematic review and meta-synthesis of qualitative data. Eur J Prev Cardiol.

[26] Resurreccion DM, Motrico E, Rigabert A, Rubio-Valera M, Conejo-Ceron S, Pastor L, Moreno-Peral P (2017). Barriers for nonparticipation and dropout of women in cardiac rehabilitation programs: a systematic review. J Womens Health (Larchmt).

[27] Worcester MU, Murphy BM, Mee VK, Roberts SB, Goble AJ (2004). Cardiac rehabilitation programmes: predictors of non-attendance and drop-out. Eur J Cardiovasc Prev Rehabil.

