# Correction to: Factors associated with non-attendance at exercise-based cardiac rehabilitation

**DOI:** 10.1186/s13102-019-0135-7

**Published:** 2019-10-17

**Authors:** Sabina Borg, Birgitta Öberg, Margret Leosdottir, Daniel Lindholm, Lennart Nilsson, Maria Bäck

**Affiliations:** 1 Department of Medical and Health Sciences, Division of Physiotherapy, Linköping University, SE-581 83 Linköping, Sweden; 2 Department of Cardiology and Department of Medical and Health Sciences, Linköping University, Linköping, Sweden; 3 Department of Clinical Sciences Malmö, Faculty of Medicine, Lund University, Malmö, Sweden; 4 Department of Cardiology, Skåne University Hospital, Malmö, Sweden; 5 Department of Medical Sciences Cardiology, Uppsala University; and Uppsala Clinical Research Center, Uppsala, Sweden; 6 Department of Medical and Health Sciences, Division of Cardiovascular Medicine, Linköping University, Linköping, Sweden; 7 Department of Occupational Therapy and Physiotherapy, Sahlgrenska University Hospital, Gothenburg, Sweden

**Correction to: BMC Sports Sci Med Rehabil(2019)11:13**

**https://doi.org/10.1186/s13102-019-0125-9**

Following publication of the original article [1], the authors reported that the family name of Daniel Lindholm was incorrectly published.

Correct: Daniel Lindholm.

Incorrect: Daniel Lindolm.
